# Swimming Exercise Promotes Post-injury Axon Regeneration and Functional Restoration through AMPK

**DOI:** 10.1523/ENEURO.0414-20.2021

**Published:** 2021-06-15

**Authors:** Sandeep Kumar, Sibaram Behera, Atrayee Basu, Shirshendu Dey, Anindya Ghosh-Roy

**Affiliations:** 1 Department of Biotechnology National Brain Research Centre, Manesar 122052, India; 2 Bruker India Scientific Pvt. Ltd, New Delhi 110019, India

**Keywords:** C. elegans, axotomy, PLM neuron, swimming exercise, axon regeneration, AAK-2

## Abstract

Restoration of lost function following a nervous system injury is limited in adulthood as the regenerative capacity of nervous system declines with age. Pharmacological approaches have not been very successful in alleviating the consequences of nervous system injury. On the contrary, physical activity and rehabilitation interventions are often beneficial to improve the health conditions in the patients with neuronal injuries. Using touch neuron circuit of *Caenorhabditis elegans*, we investigated the role of physical exercise in the improvement of functional restoration after axotomy. We found that a swimming session of 90 min following the axotomy of posterior lateral microtubule (PLM) neuron can improve functional recovery in larval and adult stage animals. In older age, multiple exercise sessions were required to enhance the functional recovery. Genetic analysis of axon regeneration mutants showed that exercise-mediated enhancement of functional recovery depends on the ability of axon to regenerate. Exercise promotes early initiation of regrowth, self-fusion of proximal and distal ends, as well as postregrowth enhancement of function. We further found that the swimming exercise promotes axon regeneration through the activity of cellular energy sensor AAK-2/AMPK in both muscle and neuron. Our study established a paradigm where systemic effects of exercise on functional regeneration could be addressed at the single neuron level.

## Significance Statement

Accelerating axonal regeneration and subsequent functional restoration is a major challenge to the people with nervous system injury. Research on rodents and humans suggests that rehabilitation therapy helps regain the lost function after neuronal injury. The nematode *Caenorhabditis elegans* provides an advantage to investigate the role of exercise in facilitating the axonal regeneration at the level of single neuron. Our study shows that swimming exercise promotes functional restoration via structural and functional changes in injured mechanosensory neuron. The benefit of exercise in regeneration depends on the metabolic energy sensor AAK-2/AMPK. This study provides a molecular perspective to exercise-mediated enhancement of axon regeneration.

## Introduction

Neuronal injuries are accompanied by physical disruption of the axons, which leads to the loss of sensory or motor function (Johnson et al., 2013; Yang et al., 2015). CNS has limited capacity to regenerate because of intrinsic failure and several inhibitory factors in the environment (Huebner and Strittmatter, 2009; He and Jin, 2016; van Niekerk et al., 2016). In case of peripheral nervous system, regeneration from the injured proximal stump and subsequent growth toward the target tissues can lead to functional restoration (He and Jin, 2016; Laha et al., 2017). However, this capability declines with age, resulting in partial or no functional restoration (Verdú et al., 2000; Geoffroy et al., 2016; Abay et al., 2017; Basu et al., 2017). Indeed, intrinsic capacity of axonal regrowth declines with age (Verdú et al., 2000) and external microenvironment poses various challenges in long distance axon regrowth (Brosius Lutz and Barres, 2014).

Although there is comprehensive understanding of axon regeneration pathways (He and Jin, 2016; Mahar and Cavalli, 2018; Richardson and Shen, 2019), protective measures against aging-related loss of regenerative potential are rather lacking. On the contrary, accumulating evidences suggest a beneficial role of rehabilitation therapies and physical exercise in promoting functional recovery following spinal cord and other injuries in human (van Hedel and Dietz, 2010; Formento et al., 2018). Physical exercise enhances functional restoration after nervous system injury in primates and rat models as well (Capogrosso et al., 2016; Fu et al., 2016). Electrical stimulation and modulation of central pattern generator leads to dramatic increase in locomotor activity in humans and rats after spinal cord lesion (Wagner et al., 2018). The benefit of exercise is accompanied by remodeling of various parts of brain (Karssemeijer et al., 2017; Horowitz et al., 2020). Although many evidence suggest that exercise promotes regrowth of injured axon in peripheral system (Park and Höke, 2014; Gordon and English, 2016; Chen et al., 2017b), it is not completely clear whether functional improvement is the outcome of rewiring of injured axon or remodeling of spared circuitry. Also, cellular and molecular mechanisms involving exercise-mediated functional improvement after nerve injury are not clear.

*Caenorhabditis elegans* is an excellent model to investigate the cellular and molecular mechanism of axon regeneration following laser-assisted injury (Hammarlund et al., 2009; He and Jin, 2016; Richardson and Shen, 2019). Axonal injury leads to calcium influx and activates the conserved p38-MAPK pathway involving dual leucine zipper kinase (DLK)-1, which initiates the transcription of regeneration associated genes (Hammarlund et al., 2009; Ghosh-Roy et al., 2010; Nix et al., 2011). Several molecular pathways controlling axon regrowth potential are identified using mechanosensory and motor neurons as model systems (Byrne and Hammarlund, 2017; Hisamoto and Matsumoto, 2017). The two posterior lateral microtubule (PLM) neurons and the DA9 motor neurons allowed correlating functional restoration with axon regrowth at the single neuron level (Abay et al., 2017; Basu et al., 2017; Ding and Hammarlund, 2018). However, it has not been tested whether physical exercise would promote axon regeneration and functional recovery. In *C. elegans*, a single or multiple swimming sessions show exercise-like features (Laranjeiro et al., 2017, 2019). Swimming sessions extend neuromuscular and gut health span, enhance learning ability, and protects against neurodegeneration (Laranjeiro et al., 2019). Exercise in an electrotactic flow chamber ameliorates the age-related degeneration (Chuang et al., 2016).

In this study, we have investigated the role of swimming exercise in improving functional restoration after axotomy of the PLM neurons. We found that a single swim session of 90 min could improve functional restoration through axon regeneration process regardless of age. This exercise regimen can also improve the age-related decline in touch response function. To understand how swimming exercise promotes functional restoration, we imaged and correlated the anatomic pattern of regrowth to the recovery index of function. This revealed that exercise promotes regrowth, self-fusion events, and postregrowth functional recovery. Improvement in functional restoration on swimming depends on the function of the cellular energy sensor AMP kinase-2/AAK-2 in both neurons and muscle.

## Materials and Methods

### *C. elegans* strains

All the strains were grown and maintained at 20°C in nematode growth media (NGM) under standard conditions (Brenner, 1974). We used the following strains: Bristol N2, *aak-2(ok524) X*, *mlk-1(ok2471) V*, *dlk-1(tm4024) I*, *ebp-1(tm1357) V*, and *unc-54(r293) I*. The extrachromosomal DNA-containing strains used were *aak-2 (ok524); shrEx362* (P*mec-4::aak-2*), *aak-2(ok524); shrEx364* (P*myo-3::aak-2*), and *aak-2(ok524); shrEX420* (P*dpy-7::aak-2*). First, the extrachromosomal arrays were obtained in *Pmec-7::GFP (muIs32)* background by injecting the rescue transgenes at 10 ng/μl. Then the transgenes were introduced into the *aak-2* mutant backgrounds by crossing. Homozygosity for all mutations was confirmed by either PCR or sequencing. All loss of function mutations are denoted as *(0)*. We used the following transgenes: *Pmec-7::GFP (muIs32), Pmec-4::GFP (zdIs5)* (Basu et al., 2017) and *Pmec-4::mcherry::RAB-3 (tbIs227)* (Sood et al., 2018).

### Age synchronization of worms

Fifty gravid adults were transferred to fresh NGM plates seeded with OP50 for egg-laying and kept at 20°C for 2–3 h. Worms were removed from the plates after they had laid eggs. The eggs were allowed to hatch and after 2 d, 40–50 L4 worms were transferred to a fresh NGM plate containing 50 μm 5-Fluoro deoxyuridine (FUDR; Sigma; catalog #F0503; Basu et al., 2017). The worms at different life stages were used for experiments.

### Swimming exercise paradigm

**Figure 1.**
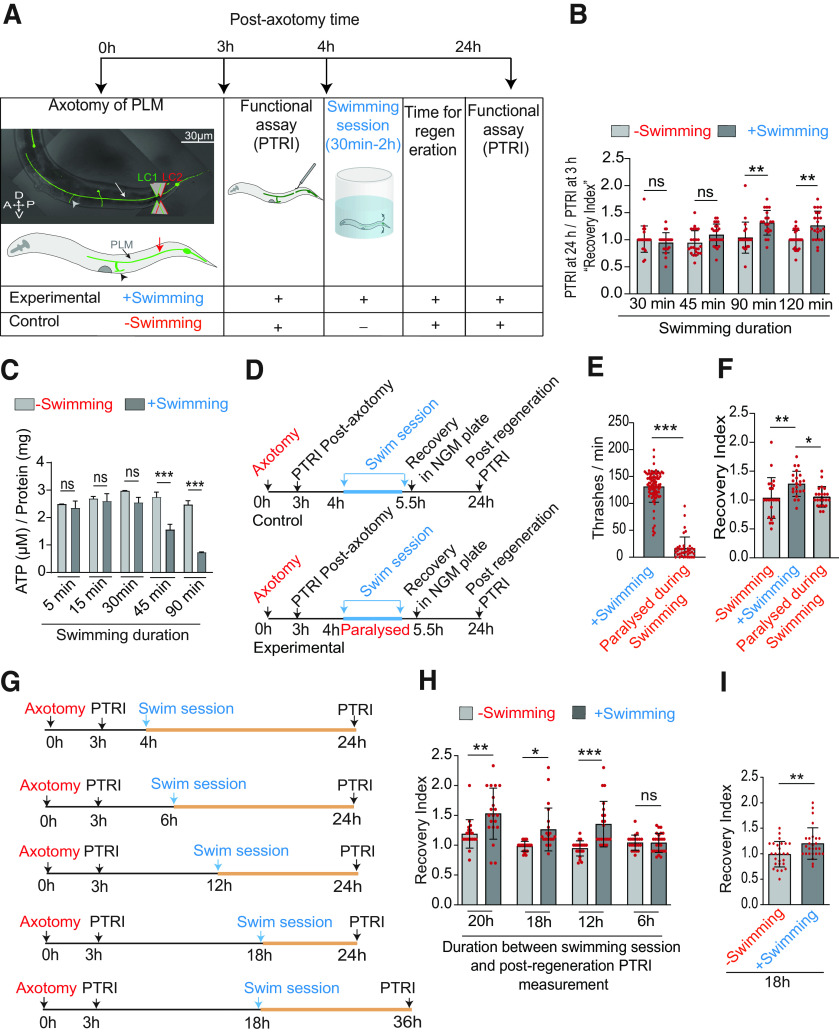
A single swim session after axotomy of PLM neurons enhances functional restoration in adult worm. ***A***, Experimental paradigm of swim session after the axotomy of a PLM neuron in A3 worms expressing P*mec-7::*GFP (*muIs32*) reporter. Following axotomy, the gentle touch response assay was performed to measure the reduction in PTRI (Extended Data Figure 1-1*A*). Then the animals were subjected to a swimming session for a duration ranging from 30 min to 2 h and recovered in NGM plate for further analysis. Another touch response assay was performed at 24 h postaxotomy to assess the functional recovery. Arrowhead denotes developmental synapses of PLM neurons. ***B***, Quantification of the recovery index expressed as PTRI at 24 h postaxotomy/PTRI at 3 h postaxotomy for swim session of varying duration. *N* = 4–5 independent replicates, *n* = 20–25 number of animals tested. The raw data used for calculating recovery index values are given in Extended Data Figure 1-2. ***C***, The bar graph represents the ATP levels measured from the total extract prepared from 60 A3-stage animals after the swim session of varying duration, *N* = 4 independent replicates. ***D***, Schematics for paralyzing the animals during the swim session. In order to prevent the worms from swimming in the well, they were treated with 5 mm levamisole for 15 s before the swimming session. The brief exposure to levamisole did not affect basal level PTRI (Extended Data Figure 1-1*B*). ***E***, The bar graph represents the thrashing frequencies measured from the time-lapse imaging of the worms in the swimming well. *N* = 3–4, *n* = 38–86. ***F***, Recovery indices obtained at 24 h postaxotomy for control and paralyzed worms during the swim session, *N* = 3–4, *n* = 22. ***G***, Scheme for determining the critical time required for seeing the beneficial effect of the swim session. In this experiment, the duration between swim session and postregeneration PTRI measurement was varied. ***H***, ***I***, Bar graphs showing the recovery indices measured according to the experimental paradigm described in ***G*** at 24 h postaxotomy and 36 h postaxotomy for ***H***, ***I***, respectively, *N* = 3–5, *n* = 20–27. Statistics, for ***B***, ***C***, ***F***, ***H***, *p < 0.05, **p < 0.01, ***p < 0.001 ANOVA with Tukey’s multiple comparison test. For ***E***, ***I***, **p < 0.01, ***p < 0.001; unpaired *t* test. Error bars represent SD; ns, not significant.

**Figure 2.**
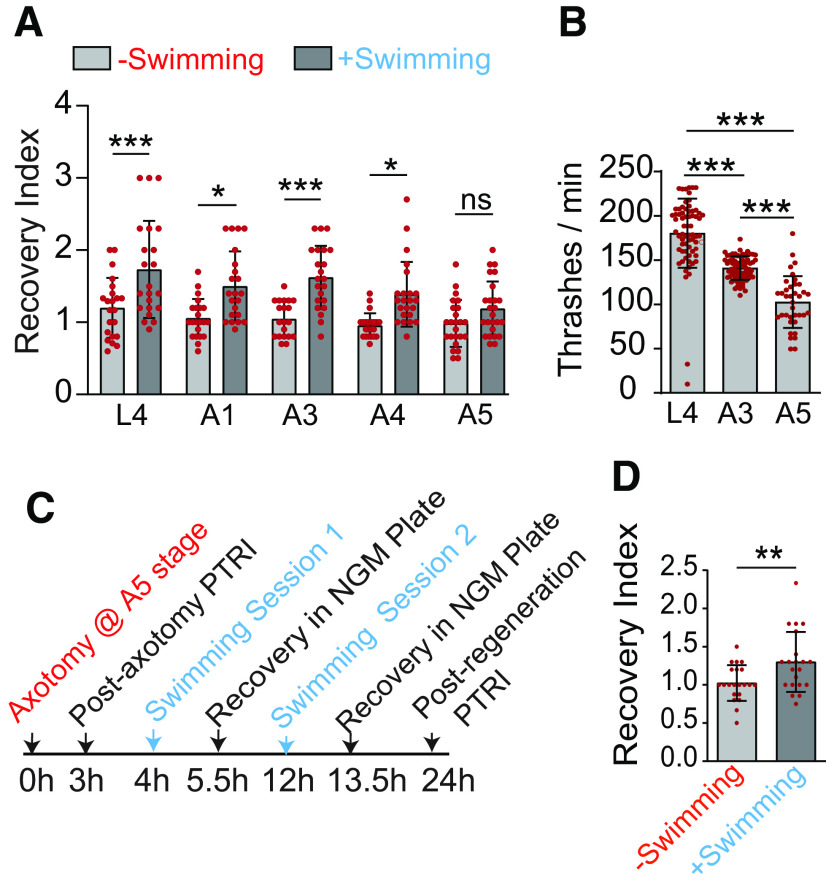
Multiple swimming-sessions after axotomy are critical for functional recovery in older ages. ***A***, The scatter plot with bars showing the effect of swimming exercise on functional recovery in L4 to A5 stage. The experiment was done in the transgenic background P*mec-7::*GFP (*muIs32*), *N* = 4–5 independent replicates, *n* = 20–25 number of animals tested. The raw data used for calculating recovery index values are given in Extended Data Figure 2-1. ***B***, Thrashing frequency of worms during the swim session at different life stages *N* = 3, *n* = 37–78. ***C***, Scheme for determining the effect of multiple swim sessions on functional recovery at A5 stage. Worms were allowed to swim for two sessions of 90 min each. ***D***, Recovery Indices in worms at 24 h postaxotomy, which underwent multiple swimming sessions after axotomy at A5 stage as shown in C-panel, *N* = 3, *n* = 21. Statistics, for ***A***, ***B***, *p < 0.05, **p < 0.01, ***p < 0.001, ANOVA with Tukey’s multiple comparison test. For ***D***, **p < 0.01, unpaired *t* test. Error bars represent SD; ns, not significant.

A single swimming session is considered as acute exercise paradigm in *C. elegans* (Laranjeiro et al., 2017, 2019). In our study, we have adopted mostly the single swim-session paradigm for exercise after axotomy of PLM neurons (Fig. 1*A*; Movie 1). Occasionally, the worms were subjected to multiple swim sessions (Fig. 2*C*). The worms were subjected to swimming in M9 buffer in 96-well plate for the duration ranging from 15 to 120 min. Single animals were kept in wells containing 200 μl of M9 buffer (one worm per well). After the desired duration of swimming, the animals were recovered on NGM plate. In the control group, worms were kept in a plate without OP50 food for the same duration and then returned to plates containing food. We found that the ATP level is significantly dropped as reported before (Chaudhari and Kipreos, 2017) after a 90-min swim session and therefore we used this 90-min session for most of the experiments.

10.1523/ENEURO.0414-20.2021.f1-1 Extended Data Figure 1-1 (A) Bar graph with scatter plot showing the PTRI before axotomy and at 3 and 24 h postaxotomy. This analysis is presented for L4 and A3 stage animals expressing *Pmec-7*::GFP (*muIs32*) reporter. *N* = 4 independent replicates, *n* = 22–31 number of worms tested. ***B***, Bar graph showing the effect of levamisole treatment on PTRI at A3 stage animals expressing P*mec-7::*GFP (*muIs32*) reporter. The worms were treated with different concentrations of levamisole for 15 s and then kept in the swimming well for 90 min. The PTRI was measured at 24 h postlevamisole treatment, *N* = 3, *n* = 14–17. Statistics, for ***A***, ***B***, ***p < 0.001; ANOVA with Tukey’s multiple comparison test. Error bars represent SD; ns, not significant Download Figure 1-1, EPS file.

10.1523/ENEURO.0414-20.2021.f1-2 Extended Data Figure 1-2 The PTRI values at 3 and 24 h postaxotomy at A3 stage. The recovery index values were obtained by normalizing the PTRI values at 24 h with respect to that at 3 h. The data are presented from groups, which underwent swimming session of varying duration (30–120 min) Download Figure 1-2, DOCX file.

### Measurement of ATP level after swim session

To measure the change in the ATP level following swim exercise, we used an ATP bioluminescence assay kit CLSII (Roche Diagnostics, catalog #11699695001; Palikaras and Tavernarakis, 2016). Briefly, 60 A3 worms from “non-swimming” as well as the “swimming” group were collected in 50 μl of M9 buffer in a 1.5-ml tube. The samples were then frozen in liquid nitrogen. The frozen tubes were kept in boiling water for 15 min. The samples were centrifuged at 14,800 × *g* for 10 min at 4°C. The supernatants were transferred to a fresh tube and diluted tenfold by adding water before measurement. The ATP levels were determined using Glomax luminometer (Promega). Before measurement, 100 μl of sample or ATP standard was added to 100 μl of luciferase in the well and incubated for 10 s at room temperature and then the luminescence was measured. The ATP level in the worm sample was derived from the ATP standard curve. Finally, the ATP levels were normalized with respect to the total protein (mg) measured through BCA protein estimation method.

### Femtosecond laser, axotomy, and imaging

For axotomy of PLM neurons, the animals were immobilized using 0.1-μm polystyrene beads on a 5% agarose pad under a coverslip. For all the experiments, only one PLM axon corresponding to either left or right side of the animal was axotomized at a distance of 50–60 μm from the cell body, as described before (Basu et al., 2017). The side corresponding to the axotomized PLM neuron is called “cut side” and other called the “control side.” Simultaneous two-photon imaging and axotomy was performed according to the previous published protocol (Basu et al., 2017). The PLM axon was imaged and axotomized with 920- and 720-nm lasers, respectively, under a 60× (Olympus) water-immersion objective of 1.1 NA on a two-photon microscope (Basu et al., 2017). This system has two tunable (wavelength range 690–1040 nm), automated depression compensated femtosecond lasers from Spectra Physics (Mai Tai with Deepsee). The imaging was done with a 6-mm galvanometer scanning system and axotomy was performed with a 3-mm galvanometer system.

### Gentle touch assay

**Figure 3.**
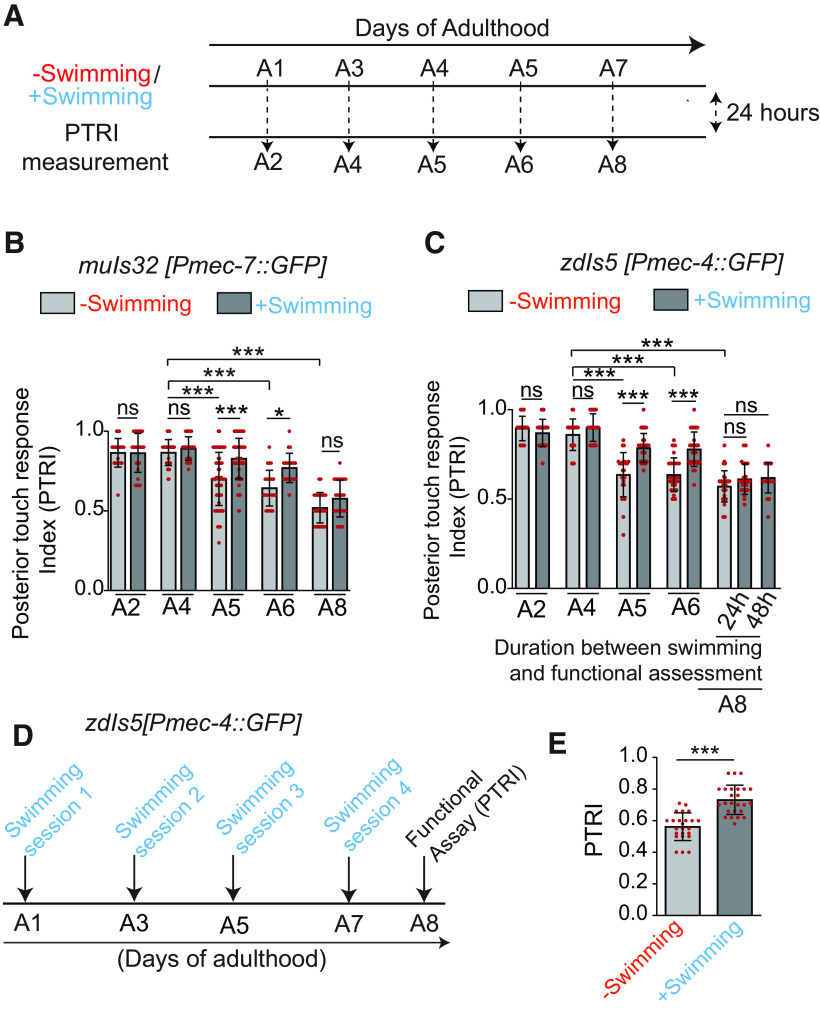
Swimming exercise prevents age-related decline in touch neuron function. ***A***, A paradigm to study the effect of swimming exercise of 90-min duration on age-dependent decline of posterior gentle touch response. Touch response assay was performed at 24 h postswimming session. ***B***, ***C***, Bar plots show the PTRIs measured at 24 h postswimming session at various life stages in two different reporter backgrounds, P*mec-7::*GFP (*muIs32*; ***B***) and P*mec-4*::GFP (*zdIs5*; ***C***). In *zdIs5* background, an additional single swim session was performed at 48 h before PTRI measurement at A8 stage; *N* = 3–5 independent replicates, *n* = 20–72 number of worms tested. ***D***, Scheme for multiple swimming sessions to enhance touch response at A8 stage. ***E***, The effect of multiple swim sessions on PTRI values at A8 stage, *N* = 3, *n* = 24–25. Statistics, For ***B***, ***C***, *p < 0.05, **p < 0.01, ***p < 0.001 ANOVA with Tukey’s multiple comparison test. For ***E***, ***p < 0.001, unpaired *t* test. Error bars represent SD; ns, not significant.

Each worm was recovered after axotomy on NGM plate and then gentle touch assay was performed. Gentle Touch Response (Chalfie and Sulston, 1981; Chalfie et al., 1985; Basu et al., 2017) was assayed from both right and left sides of the worm. Following the touch assay in one side, the worm was flipped and kept for 20 min before touching the other side (Basu et al., 2017). Ten alternative anterior and posterior touches were given with the eyelash tip. The anterior touch was given to a forward-moving animal, which in response started moving backward. When a backward moving animal was given a posterior touch, it started moving forward in response. A positive response was denoted as 1, and no response as 0. Then the posterior touch response index (PTRI) was measured as the ratio of total number of positive response to total number of touch stimuli applied (Extended Data Fig. 1-1, Fig. 3*B-C*) as described before (Basu et al., 2017).

### Correlation of functional recovery with axon regeneration events at the level of a single worm

**Figure 4.**
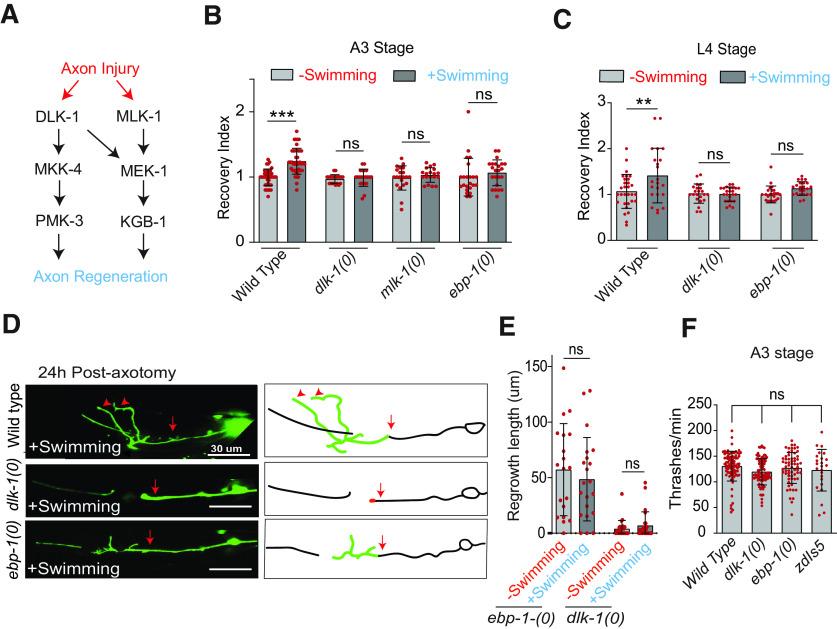
Swimming session-related improvement in functional recovery is dependent on axon regeneration. ***A***, Pathway diagram of two parallel p38-MAPK pathways involving DLK-1 and MLK-1 in axon regeneration. ***B***, The quantification showing the effect of swimming exercise on the recovery index measured at 24 h postaxotomy in the *dlk-1(0)*, *mlk-1(0)*, and *ebp-1(0)* at A3 stage. *N* = 3–4 independent replicates, *n* = 19–42 number of worms tested. ***C***, The recovery indices measured at 24 h postaxotomy in the *dlk-1(0)* and *ebp-1(0)* at L4 stage with and without swimming conditions *N* = 3–5, *n* = 22–30. ***D***, ***E***, Representative confocal images of PLM axons at 24 h postaxotomy in the wild-type control and mutants affecting axon regeneration pathways (***D***) and axon regrowth values (***E***) measured from the proximal cut tips (arrows in ***D***), *N* = 3–4, *n* = 19–25. Red arrowheads represent the filipodia-like structure at the regrowing tips, and arrows indicate the position of axotomy. ***F***, Thrashing frequencies in the mutants at A3 stage *N* = 3–4, *n* = 24–85. Statistics, for ***B***, ***C***, ***E***, ***F***, **p < 0.01, ***p < 0.001; ANOVA with Tukey’s multiple comparison test. Error bars represent SD; ns, not significant.

**Figure 5.**
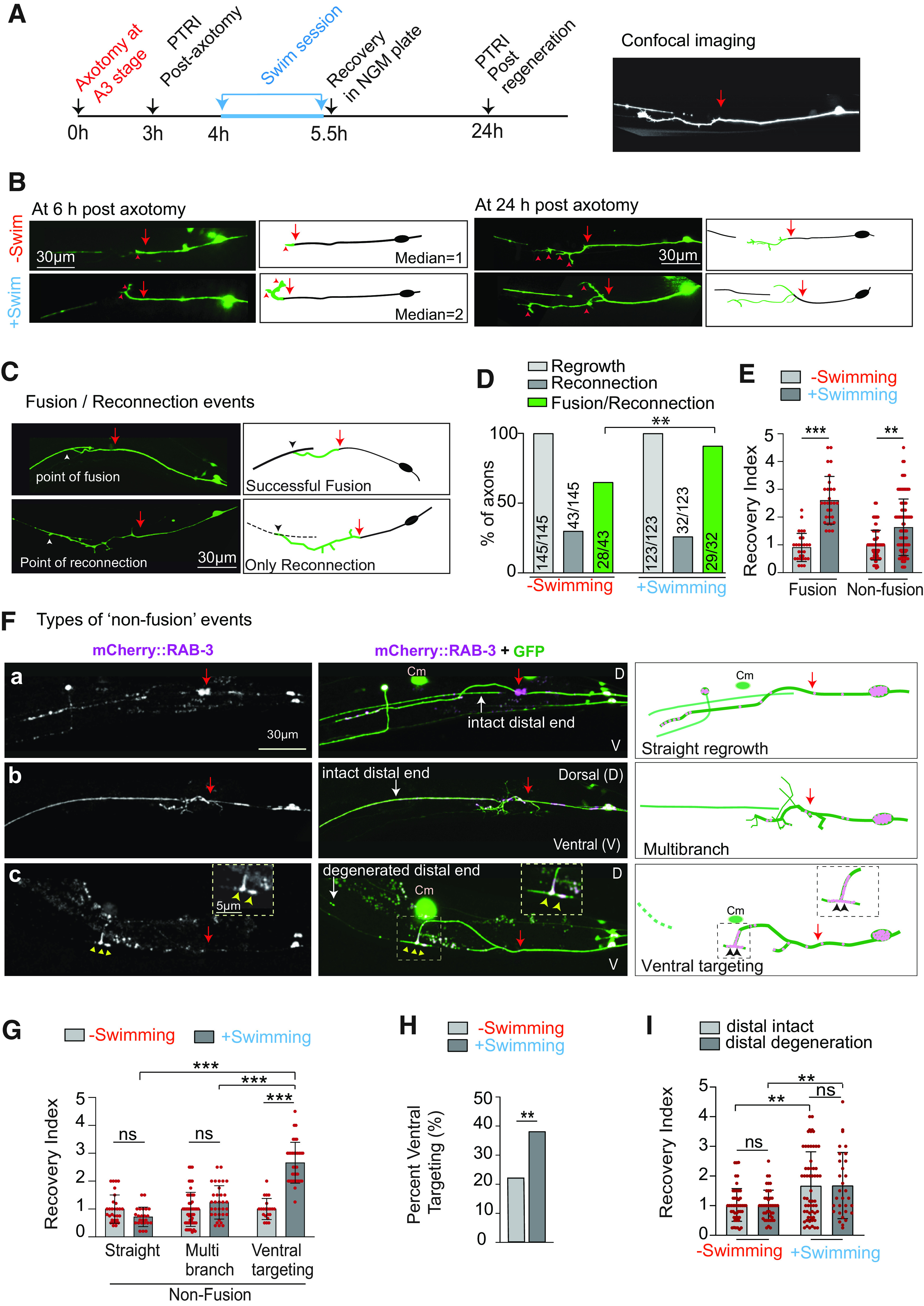
Swimming exercise promotes both axon regrowth and the functional recovery. ***A***, Experimental design to correlate functional recovery with the anatomic features of axon regrowth in worms expressing P*mec-7*::GFP (*muIs32*) at A3 stage. The confocal imaging was done after the measurement of PTRI values at the 24 h postaxotomy. ***B***, Representative images and illustration of PLM axons from control and swimming group at 6 and 24 h postaxotomy. Arrows and arrowheads indicate the position of axotomy and filopodia-like structures, respectively. ***C***, Confocal images of fusion and “reconnection” events. Arrows indicate the position of axotomy. ***D***, Bar graph representing the % of different types of regeneration events in swimming and non-swimming groups, *N* = 3–5 independent replicates, *n* = 123–145 neurons. ***E***, Bar graph with scatter plots presenting changes in the recovery index corresponding to the fusion and non-fusion events because of swim session. *N* = 3–5, *n* = 52–91 worms. ***F***, Confocal images representing different types of non-fusion regeneration events. The worms are expressing the presynaptic marker P*mec-4*::mCherry-RAB3 (*tbIs227*) and P*mec-7::*GFP (*muIs32*). Yellow dashed box showing the enrichment of RAB-3 at the ventral cord region in the ventral targeting event. Arrowheads indicate enriched RAB-3 puncta. Red arrows indicate the position of axotomy; Cm denotes coelomocyte cell expressing the co-injection marker. ***G***, Bar graph with scatter plots showing the changes in the recovery index because of swim exercise in different classes of non-fusion events, *N* = 3–5, *n* = 21–39. ***H***, The change in the % of ventral targeting events because of swimming exercise. ***I***, A comparison of recovery indices between the distal axon intact and distal axon degenerated events with respect to swimming exercise, *N* = 3–5, *n* = 31–60. Statistics, for ***E***, ***G***, ***I***, **p < 0.01, ***p < 0.001 ANOVA with Tukey’s multiple comparison test. For ***D***, ***H***, **p < 0.01 χ^2^ test. Error bars represent SD; ns, not significant.

At 3 h after axotomy, PTRIs of both axotomized and control (uncut) side, were denoted as the PTRI postaxotomy. Each worm was labeled based on the side of axotomy and kept in single plate at 20°C. After 24 h, PTRI values from both the sides were measured and compared with the corresponding values at the postaxotomy stages. For a given side, the Recovery Index was obtained by the following formula: recovery index = PTRI_24 h_/PTRI_3 h_ (Extended Data Fig. 1-2). After the behavioral test at 24 h, the regrowth pattern of the PLM axon was scored either using a Leica DM5000 fluorescent microscope at 40× magnification or a Spinning disk confocal microscope (Fig. 4*D*, Fig. 5*A*). Specifically, it was noted whether it was a fusion event (Fig. 5*C*) or non-fusion event (Fig. 5*F*). For scoring successful fusion events, we carefully evaluated whether the proximal end has just touched the distal counterpart or it has successfully joined and fused with the distal end (Fig. 5*C*). In case of casual touching, the distal end eventually undergo degeneration, and these events are referred as reconnection (Fig. 5*C*; Neumann and Hilliard, 2019).

### Imaging of axon regrowth events

At 24 h postaxotomy, for imaging of the regeneration events, the animals were immobilized using 10 mm levamisole hydrochloride. Axonal regrowth was imaged using a Zeiss 864 Axio-Observer Z1 microscope equipped with Yokogawa CSU-XA1 spinning-disk confocal scan-head and a Photometrics Evolve EMCCD camera, the images were taken using 63× oil objective of 1.46 NA. The P*mec-7*::GFP labeled PLM axon was imaged with 30% input power of 480-nm laser. The images were acquired with the exposure time of 300 ms with the 70% of camera gain settings. The images were then exported as czi files and analyzed using ImageJ software. For regrowth length measurement, simple neurite tracer plugin of ImageJ was used*. Pmec-4::mcherry::RAB-3 (tbIs227)* reporter was used to image the formation synapse-like structures during axon regeneration. Imaging was done using a Nikon A1 plus (Nikon corporation) confocal microscope. PLM neurons expressing *muIs32* and *tbIs227* reporter were simultaneously imaged after 24 h of axotomy. Imaging was done under a 60× oil objective (NA = 1.4) at 1 μm slice interval. Excitation power was 0.8 and 1 for 488- and 561-nm laser, respectively. The PMT power for 488-nm channel was 100 and 104 for 561-nm channel with offset value of 20.

Each PLM neuron has a ventral branch (Fig. 1*A*, arrowhead), which makes synapse onto the postsynaptic interneuron (Chalfie and Sulston, 1981; Chalfie et al., 1985). This allowed us to set the dorsal-ventral axis of the worm (Fig. 5*F*) while analyzing the direction of axon regrowth. The axons that regrew up to 35–45 μm in depth in ventral direction and extended along the ventral nerve cord were characterized as the “ventral targeting” event (Fig. 5*Fc*). An accumulation of the presynaptic reporter mCherry::RAB-3 along the ventral cord (Fig. 5*Fc*, yellow arrowheads) was also noticed in these events. This accumulation pattern resembled the original chemical synapses of PLM neurons. When the distal end intensity was prominently less and showed beaded appearance, it was categorized as “distal degeneration” (Fig. 5*Fc*) otherwise categorized as “distal intact” (Fig. 5*Fa,b*).

### Paralysis of the worms in swimming well

At 3 h after axotomy, the animals were treated with 5 mm levamisole hydrochloride for 15 s to paralyze them. After this brief treatment with levamisole, they were placed in swimming wells. We found that this brief treatment is sufficient to perturb the swimming (Movie 2; Fig. 1*E*) during the 90-min swim session. To verify whether this brief treatment of levamisole has any effect in the gentle touch response of the animal, we performed similar paralysis without performing axotomy and then measured the touch response after 24 h. We found that the PTRI value was not affected by this brief treatment (Extended Data Fig. 1-1*B*). Therefore, we used this experimental design to test the effect of blocking the swimming on enhancement of functional regeneration (Fig. 1*D–F*).

### Measurement of thrashing frequency during swimming

For the measurement of thrashing frequency, digital videos of worm movements were acquired during the swimming sessions, using a Leica MC 120 HD camera. The videos were recorded for 30 s at 15 frames/s using LAS V4.4 software in a Leica stereo microscope M165 FC at 1.25× magnification. The thrashing frequency were measured as body bends per second using wrMTrck plugin of ImageJ software (http://www.phage.dk/plugins/wrmtrck.html). The change in the direction of bending at the mid-body was defined as one body bend (Truong et al., 2015). A mutant for muscle myosin *unc-54* (Pulak and Anderson, 1988) showed drastically reduced thrashing frequency using this plugin. Thrashing frequency in *unc-54* mutant was 18.27 ± 13.04 as opposed to 130.8 ± 28.97 for wild type. This suggests that our analysis is sensitive enough to show the difference in swimming ability because of various experimental conditions.

### Metformin treatment on paralyzed worms

At 3 h postaxotomy, worms were first treated with 5 mm levamisole hydrochloride solution for 15 s to cause the muscle paralysis. The experimental swimming well had 50 mm Metformin hydrochloride (Sigma-Aldrich; catalog #PHR1084). Therefore, the paralyzed worms were treated with Metformin during the 90-min swim session. Concentration of Metformin were chosen to activate AMPK/AAK-2 based on the previous report in *C. elegans* (Chen et al., 2017a).

### Molecular biology and transgenes

For touch neuron, muscle and epidermal-specific expression of *aak-2*, first a gateway (Thermo Fisher Scientific) entry clone of *aak-2* [pCR8::*aak-2* (pNBRGWY115)] was constructed by PCR with the primers 5′-ATGTTTTCTCATCAAGATCGAGA-3′ and 5′-TCTCGATCTTGATGAGAAAACAT-3′. Then the entry clone was recombined with pCZGY553 (P*mec-4* destination vector), pCZGWY925 (P*myo-3* destination vector) and pCZGWY44 (P*dpy-7* destination vector) to generate P*mec-4*::*aak-2* (pNBRGWY116), *Pmyo-3*::*aak-2* (pNBRGWY117) and P*dpy-7::aak-2* (pNBRGWY149), respectively.

### Statistics

All the statistical analyses were performed using GraphPad Prism software version 9.0.2. For two-way comparisons, an unpaired *t* test with Welch’s correction was used. The median values were compared with the Mann–Whitney *U* test. Fisher’s exact test was used for proportions. Three or more samples were compared with ANOVA (nonparametric) with a *post hoc* Tukey’s multiple comparisons test. The sample numbers (*n*) presented on each bar are the total sample value accumulated over the total number of biological replicates (*N*) in a given experiment.

## Results

### A single swim session after axotomy of PLM neurons promotes functional recovery

Previous studies have indicated that physical exercise following nervous system injury promotes axon regeneration and functional recovery in various model systems (Doyle and Roberts, 2006; Asensio-Pinilla et al., 2009; Sachdeva et al., 2016; Kuwabara et al., 2018). To address whether physical exercise can enhance functional restoration in *C. elegans*, we designed a swimming exercise paradigm in conjunction with PLM axon regeneration (Fig. 1*A*; Movie 1). A single session of swimming in *C. elegans* mimics the features of mammalian exercise (Laranjeiro et al., 2017). As previously reported that there is a sharp drop in functional recovery of gentle touch behavior after the axotomy of PLM neuron at day-3 adult worms (A3; Basu et al., 2017; Extended Data Fig. 1-1*A*), we tested whether a swim session after axotomy would enhance recovery. We measured the PTRI at 3 and 24 h postaxotomy (Fig. 1*A*). The extent of functional recovery was represented as the normalized PTRI at 24 h with respect to that measured at 3 h postaxotomy, which we called “recovery index” (Fig. 1*B*; Extended Data Fig. 1-2). A recovery index value, higher than 1 is an indication of improvement of touch response over the time after axotomy. A swimming session of 90 min or above significantly enhanced the recovery index at 24 h as compared with the non-swimming control (Fig. 1*B*; Extended Data Fig. 1-2). It also correlated with the significant drop in ATP level after the swimming session measured fluorometrically from the worm lysate (Fig. 1*C*). After 90 min, the worms did episodic swimming rather than continuous swimming (Ghosh and Emmons, 2008). Therefore, we chose 90-min exercise window for our additional experiments. To confirm that the swimming-induced improvement in functional recovery is not because of the stress in liquid environment, rather because of exercise, we paralyzed the worm during swimming using levamisole that causes muscle hypercontraction (Culetto et al., 2004; Fig. 1*D*,*E*). The change in swimming ability was measured as thrashing frequency (Buckingham and Sattelle, 2009; Fig. 1*E*). We observed that the recovery index was significantly reduced when swimming was perturbed (Movie 2; Fig. 1*F*). Levamisole treatment in the swimming well per se did not affect the touch response index measured after the withdrawal of levamisole (Extended Data Fig. 1-1*B*).

To determine the time duration required for seeing the benefit of swimming, we varied the time window between the swim session and evaluation of touch response (PTRI; Fig. 1*G*). Essentially, the swim session was gradually shifted toward the time of postregeneration PTRI measurement (Fig. 1*G*). When the window was lower than 12 h, in this case 6 h, the functional improvement was non-significant (Fig. 1*H*). But when we increased the window, by shifting the time of postregeneration PTRI measurement further by 18 h, the functional improvement was significant (Fig. 1*I*). Therefore, a critical time of 12 h is needed for proper manifestation of positive effect of swimming in regeneration.

### Multiple swim sessions are needed in older ages for the improvement in functional restoration

Next, we expanded our single swim-session paradigm across various ages and found that this exercise regimen can improve the recovery index significantly when axotomy was performed at L4, A1, A3, and A4 stages (Fig. 2*A*; Extended Data Fig. 2-1). However, this improvement was non-significant at day 5 (A5; Fig. 2*A*; Extended Data Fig. 2-1). We noticed that the thrashing frequency during swimming was significantly reduced at A5 stage (Fig. 2*B*), which might have reduced the beneficial effect of swimming in functional restoration. We wanted to test whether multiple swimming sessions would be required at A5 stage for significant improvement in functional recovery. To test this hypothesis, we increased the number of swim sessions after the axotomy at A5 stage, one at 4 h and the other at 12 h postaxotomy (Fig. 2*C*). Increasing the number of swim session raised the recovery index value significantly (Fig. 2*D*). Overall, our data suggest that swimming-related exercise promotes functional restoration regardless of age, although in older age, multiple exercise sessions are important.

10.1523/ENEURO.0414-20.2021.f2-1 Extended Data Figure 2-1 The PTRI values at 3 and 24 h postaxotomy in wild-type worms at different life stages (L4–A5). The recovery index values were obtained by normalizing the PTRI values at 24 h with respect to that at 3 h. The experimental group underwent a swimming session of 90-min duration. Download Figure 2-1, DOCX file.

### Swim exercise also prevents the age-related decline in touch neuron function

It might be possible that swim exercise improves the touch neuron function in general, especially in older age when function is known to decline (Basu et al., 2017). As reported previously, we found that PTRI value is significantly dropped at A5 and subsequent life stages (Fig. 3*B*). A single swim session of 90 min, 1 d before the PTRI measurement (Fig. 3*A*) improved the PTRI value significantly at A5 and A6 stages (Fig. 3*B*,*C*). However, in A8 stage, the improvement in PTRI value because of single session swimming exercise was poor (Fig. 3*B*,*C*). No improvement at A8 stage was also noticed in the *zdIs5* (P*mec-4::*GFP) transgenic reporter background (Fig. 3*C*). We asked whether a longer gap between swim session and functional assessment would be sufficient to enhance the PTRI value significantly at A8 stage. However, no further improvement in PTRI value was noticed when the exercise session was anticipated by 1 d (Fig. 3*C*). To test whether multiple swimming sessions across multiple days are helpful, we subjected the animals to four swimming sessions starting from A1 with 1-d interval between two sessions and then measured touch response at A8 stage (Fig. 3*D*). Multiple swimming sessions significantly elevated the PTRI value at A8 stage as compared with that obtained from non-swimming control (Fig. 3*D*,*E*). Our data highlight that exercise improves both age-dependent decline in postaxotomy functional recovery, as well as prevents the age-related decline in touch neuron function. This raises the possibility that the exercise might only improve the neuronal function. Therefore, it needs to be resolved whether our exercise regimen would also enhance axon regrowth potential after axotomy.

### Swim session-mediated improvement in postaxotomy functional restoration involves initiation of axonal regeneration

Physical exercise mediated improvement in functional recovery after neuronal injury often involves remodeling of spared neuronal circuits (van den Brand et al., 2015). Therefore, we wanted to test whether the benefit of swimming is because of compensatory mechanisms or regrowth of the axotomized PLM neuron. The p38-MAPK pathway involving DLK-1/MLK-1 is required in the early stages of axon regeneration in a cell-autonomous manner (Fig. 4*A*; Hammarlund et al., 2009; Yan et al., 2009; Ghosh-Roy et al., 2010). In the absence of this signaling cascade, injured axon cannot initiate the growth cone formation after axotomy. Therefore, the mutants affecting the DLK-1 cascade serve as a tool to block the regeneration from the injured proximal stump after axotomy (Fig. 4*D*, red arrows). A swim session neither in *dlk-1(0)* nor in *mlk-1(0)* could promote functional restoration in A3 (Fig. 4*B*). Similar observation was made in *dlk-1(0)*, when the experiment was conducted at L4 stage (Fig. 4*C*). Similarly, an upregulation of microtubule dynamics through microtubule plus-end binding protein-1 EBP-1 is critical for the efficient axon regrowth (Chen et al., 2011; Ghosh-Roy et al., 2012). We found that, the benefit of exercise was not seen in the absence of *ebp-1* (Fig. 4*B*,*C*). Consistently, swimming could not promote the axon regrowth from the cut stump in these mutants (Fig. 4*D*,*E*, red arrows). To rule out the possibility of reduced swimming ability in these mutants, we analyzed the movies of their swimming. We found that thrashing frequency in these mutants are comparable to that in wild-type control (Fig. 4*F*).

These results confirm that exercise mediated increase in functional recovery involves regrowth from the injured proximal stump of the PLM neuron and subsequent rewiring into the functional circuit.

### Swim session promotes axon regrowth and postregrowth functional recovery

Although swimming induced improvement in functional restoration involves initiation of axon regrowth after axotomy, it is not clear whether this exercise paradigm would enhance anatomic features of axon regrowth or it could simply enhance the functional aspect after axon regrows. The fact that swimming can enhance touch sensation behavior in older age, leaves the second possibility open. To test this, we performed confocal imaging of the regrowth events after assaying for the functional restoration (Fig. 5*A*), and correlated the behavioral recovery with the anatomic patterns of regeneration. We found that swimming exercise in A3 worms accelerated the initiation of the regrowth as revealed by the increased number of filopodia-like extension at the cut stump at 6 h postaxotomy (Fig. 5*B*, arrowheads). The median value for the number of filopodia is increased from 1 to 2 because of the exercise session (*p = 0.01, Mann–Whitney *U* test). At 24 h postaxotomy, there was an enhancement in axonal regrowth as compared with the control condition (Fig. 5*B*). Regrowth value in the swimming group becomes 114.0 ± 63.81 μm as compared with the value 83.46 ± 41.62 μm obtained in non-swimming group (*p = 0.04, unpaired *t* test). We also noticed that the percentage of self-fusion events with respect to the reconnection events (Ghosh-Roy et al., 2010; Neumann et al., 2011, 2015; Basu et al., 2017; Neumann and Hilliard, 2019) between proximal and distal ends are significantly increased because of the swimming session (Fig. 5*C*,*D*).

We further investigated whether the improvement in functional restoration seen because of swim exercise correlates with the “fusion” or “non-fusion” events, or both the categories of regrowth. We found that there is a significant increase in the recovery index for both the fusion event and non-fusion events (Fig. 5*E*). Upon correlating the regrowth pattern of the non-fusion events, we found that the regrowing axon, which goes toward the ventral cord and shows an enrichment of the presynaptic reporter mCherry::RAB-3 at the ventral cord (Fig. 5*Fc*, arrowheads) corresponds to successful recovery in the swimming group as the value of the recovery index of this class is 2.66 + 0.73 (Fig. 5*G*). We called this category of as ventral targeting events. The recovery index corresponding to the ventral targeting events was significantly higher as compared with the index obtained in the other classes such as “straight regrowth” (Fig. 5*Fa*) and “multibranch regrowth” (Fig. 5*Fb*). The percentage of ventral targeting events got increased on swim exercise (Fig. 5*H*). Since the distal end often persisted after injury (Fig. 5*Fa*,*b*), we asked whether the “intact distal end” could be contributing to the functional recovery. However, the recovery indices in the distal intact and “distal degenerated” categories were comparable in both swimming and non-swimming groups (Fig. 5*I*). Therefore, the enhancement of functional restoration because of swim exercise corresponds to successful rewiring process.

### Exercise-mediated improvement in touch neuron function requires metabolic energy sensor AAK-2

**Figure 6.**
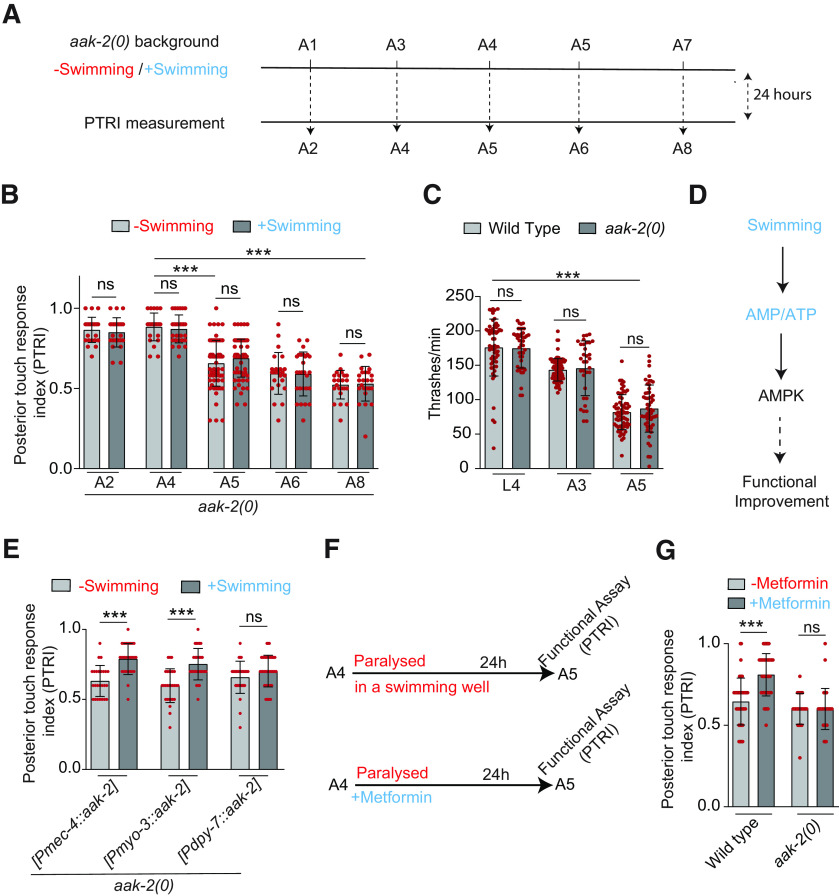
Swimming mediated improvement in touch response in older age is dependent on AAK-2. ***A***, Schematic to study the effect of single swimming session on age- related decline in touch neurons function in *aak-2* mutant. Touch response assay was performed 24 h postswimming session. ***B***, The PTRIs measured at 24 h postswimming session in *aak-2(0)* at different life stages, *N* = 4–6 independent replicates, *n* = 23–68 number of animals. ***C***, The thrashing frequencies (body bends/min) in wild type and *aak-2(0)* at larval (L4) and adult stages, *N* = 3–4, *n* = 33–90. ***D***, A pathway diagram explaining how a swim session might enhance touch neuron function through AMPK. ***E***, The effect of swimming exercise on the decline in touch response at A5 stage in *aak-2(0)* expressing *Pmec-4::aak-2 (shrEx362)*, *Pmyo-3::aak-2 (shrEx364)*, and *Pdpy-7::aak-2(shrEx420)* transgenes, *N* = 3, *n* = 25–41. ***F***, Schematics of metformin treatment to the paralyzed worms in the swimming well to study the effect of AMPK activation in touch neuron function at A5 stage. The wild-type and *aak-2(0)* worms were pretreated with 5 mm levamisole for 15 s before the swimming session. ***G***, Changes in PTRI values at at 24 h posttreatment with 50 mm metformin as shown in ***F***, *N* = 5, *n* = 28–51. Statistics, for ***B***, ***C***, ***p < 0.001; ANOVA with Tukey’s multiple comparison test. For ***E***, ***G***, ***p < 0.001; unpaired *t* test. Error bars represent SD; ns, not significant.

During exercise, there is a consumption of energy in the form of ATP, which results in an increase in an AMP:ATP ratio (Chen et al., 2003). The increase in this ratio is sensed by the metabolic energy sensor kinase known as AMPK (Hardie, 2011). The physical exercise in mammalian system leads to activation of AMPK (Chen et al., 2003; Gibala et al., 2009). As in mammals, the two catalytic subunits of AMPK in *C. elegans* are encoded by two genes *aak-1* and *aak-2* (Apfeld et al., 2004). We hypothesized that the decrease in ATP levels after the 90-min swimming session might be sensed by AAK-2 in neurons or muscle to regulate regeneration. To test this possibility, we gave the *aak-2* mutant a 90-min swim session at various life stages starting from A1 to A7 and measured the posterior touch response (PTRI) after 24 h (Fig. 6*A*). We found that there is drop in touch response index value in *aak-2* mutant starting from A5 stage similar to the wild type (Fig. 6*B*). However, the swim session could not elevate the PTRI in any of the life stages (Fig. 6*B*). We wondered, whether the abrogation of the exercise-induced elevation in PTRI value in *aak-2(0)* is due the reduced swimming ability of the mutant. However, the thrashing frequency in this mutant at L4, A3, and A5 stages were comparable to the same values in wild-type control (Fig. 6*C*). This indicated that AAK-2 might be specifically required for the exercise-induced changes in neuronal regeneration and function (Fig. 6*D*). To understand the tissue-specific requirement of *aak-2* in this phenomenon, we have expressed *aak-2* in muscle, epidermal cells and touch neuron exclusively. We found that both touch neuron and muscle-specific expression of *aak-2* can rescue the swim session-induced phenomenon significantly (Fig. 6*E*). However, no rescue was observed when *aak-2* was expressed in epithelial cells situated just next to the PLM axon (Fig. 6*E*). Next, we tested whether the AMPK activator metformin (Foretz et al., 2014; Rena et al., 2017) can mimic the benefits of swimming in touch neuron function (Fig. 6*F*). To address this question, we treated the worms with 50 mm metformin while they were kept paralyzed in the swimming well for 90 min (Fig. 6*F*). Metformin treatment was sufficient to enhance the PTRI value in A5 worms (Fig. 6*G*). This effect of metformin was absent in *aak-2* mutant (Fig. 6*G*) indicating that effect of metformin in our assays is specific to AAK-2 function. These observations suggest that neuron-specific and muscle-specific activity of AAK-2 is important for swim-exercise-induced enhancement of touch neuron function.

### Activation of the energy sensor AAK-2 after axotomy is sufficient to promote axon regrowth and functional restoration

**Figure 7.**
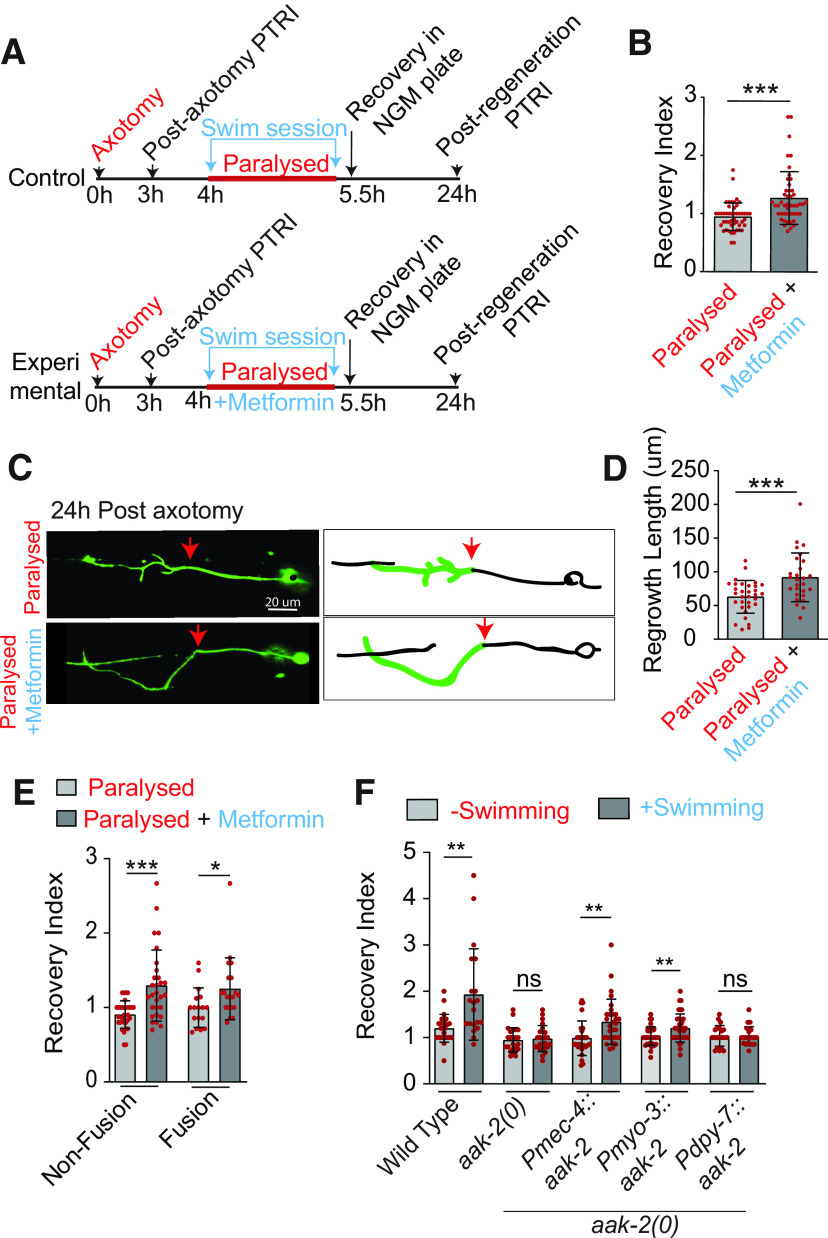
Swimming-mediated improvement in functional recovery after axon regeneration involves the energy sensor AAK-2. ***A***, Strategy for the AMPK activation during the swim session by treating with metformin. Wild-type worms were pretreated with 5 mm levamisole for 15 s before the swimming session to paralyze them. 50 mm metformin was applied to the paralyzed worms in the swimming well. ***B***, The effect of metformin treatment on recovery index at 24 h postaxotomy at A3 stage *N* = 5 independent replicates, *n* = 47–48 number of worms. ***C***, ***D***, Representative confocal images (***C***) and quantification (***D***) of PLM axon regrowth at 24 h postinjury in wild-type worms treated with or without metformin after axotomy at A3 stage. *N* = 5, *n* = 27–32. Arrows indicate the position of axotomy. ***E***, Bar graph represents the comparison of recovery index in control and metformin-treated worms at A3 stage, *N* = 3, *n* = 48–49. ***F***, The effect of touch neuron, muscle and epidermal cell-specific expression of *aak-2* in *aak-2* mutant on swimming induced enhancement in recovery index. The rescue transgenes P*mec-4::aak-2* (*shrEx362*), P*myo-3::aak-2* (*shrEx364*), and P*dpy-7::aak-2* (*shrEx420*) were used, *N* = 3, *n* = 20–37. The regrowth length and basal level of recovery index values in *aak-2(0)* mutant with or without the transgenes of *aak-2* at L4 stage is presented in Extended Data Figure 7-1. Statistics, for, ***B***, ***D***, ***E***, ***F***, *p < 0.05, **p < 0.01, ***p < 0.001 unpaired *t* test. Error bars represent SD; ns, not significant.

To address the role of AMPK/AAK-2 during swimming, we treated the 3-d-old (A3) worms at 3 h postaxotomy with 50 mm metformin only during the 90-min swim session, while these animals were paralyzed with levamisole in the swimming well (Fig. 7*A*). Previously we have shown that when worms are paralyzed during the swim session, enhancement of functional restoration is blocked (Fig. 1*F*). We found that metformin treatment significantly enhanced the recovery index as compared with the non-treated control (Fig. 7*B*). Total regrowth from the proximal stump (Fig. 7*C*, red arrows) at 24 h postaxotomy was also increased significantly in the metformin-treated group (Fig. 7*C*,*D*). The regrowth value becomes 91.77 ± 36.19 μm in metformin-treated group as compared with 62.87 ± 24.36 μm in untreated group. We noticed that there is an increase in the recovery index for both non-fusion-related and fusion-related events at A3 stage (Fig. 7*E*). These observations suggest that AMPK activation is sufficient to mimic the effect of swimming exercise in axonal regeneration as well as functional recovery.

10.1523/ENEURO.0414-20.2021.f7-1 Extended Data Figure 7-1 ***A***, Confocal images of a PLM neurons labelled with *Pmec-7::GFP (muIs32)* in *aak-2(0) and* tissue-specific rescue background at 24 h postaxotomy at the L4 stage. Red arrows indicate the position of axotomy. ***B***, Rescue of axon regrowth values in *aak-2(0)* at 24 h postaxotomy by the touch neuron, muscle and epidermal cell-specific expression of *aak-2* cDNA. *N* = 3–4, *n* = 17–30 animals. ***C***, The rescue of recovery index at 24 h postaxotomy in *aak-2(0)* by the touch neuron, muscle and epidermal cell-specific expression of *aak-2* cDNA. Rescue transgenes P*mec-4*::*aak-2* (*shrEx362*), P*myo-3*::*aak-2* (*shrEx364*), and P*dpy-7*::*aak-2* (*shrEx420*) were used, *N* = 2–4, *n* = 21–26. Statistics, for ***A***, ***C***, ***D***, *p < 0.05, **p < 0.01, ***p < 0.001; unpaired *t* test; *n* = number of worms tested. Error bars represent SD; ns, not significant. Download Figure 7-1, EPS file.

Movie 1. After the measurement of PTRI, worms were transferred to wells and allowed to swim for 90 min. The videos were recorded at 15 frames/s (fps) and represented at 50 fps. 10.1523/ENEURO.0414-20.2021.video.1

Movie 2. Swimming of control and paralyzed worms. In order to paralyze the worms, the worms were treated with 5 mm levamisole and transferred to the swimming well. Movies were acquired at 15 frames/s (fps) and represented at 50 fps. 10.1523/ENEURO.0414-20.2021.video.2

The *aak-2* mutant shows reduced axon regrowth (Hubert et al., 2014; Extended Data Fig. 7-1*A*,*B*), which resulted in a loss of swim-exercise-mediated enhancement of postaxotomy recovery index at A3 stage (Fig. 7*F*). We found that the expression of *aak-2* either in touch neurons or in muscle rescues the axon regrowth defect in *aak-2* mutant at L4 stage (Extended Data Fig. 7-1*A*,*B*). However, the postinjury recovery index was only rescued significantly by the touch neuron-specific expression of *aak-2* at L4 stage (Extended Data Fig. 7-1*C*). These transgenes also rescued the loss in benefit of swim session in functional recovery in *aak-2* mutant at A3 stage (Fig. 7*F*). Expression of *aak-2* in epithelial cells however did not rescue these phenotypes in *aak-2* mutant (Fig. 7*F*; Extended Data Fig. 7-1*A–C*). These results indicate that AAK-2 acts both cell autonomously in neuron and non-cell autonomously in muscle for transducing the effect of swim exercise in effective axon regeneration.

## Discussion

**Figure 8.**
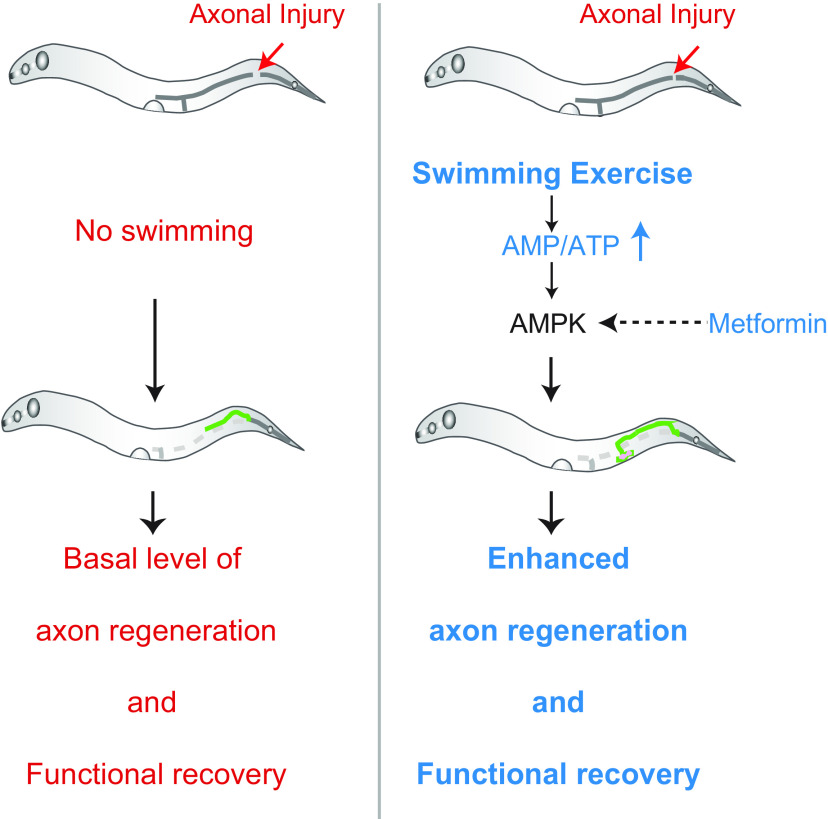
Proposed model illustrating how swimming exercise promotes axon regeneration. Exercise session after axonal injury leads to the consumption of cellular ATP resulting in activation of AMPK. An activated form of AMPK promotes axon regeneration and functional restoration.

In this study, using a well-established swimming paradigm we have studied the effect of physical exercise in functional recovery after the axonal injury of mechanosensory neuron. A swim session following axotomy promotes functional recovery regardless of age. This exercise regimen can also help overcome age-related decline in touch neuron function. By correlating anatomic features of axon regrowth with the behavioral index, we found that the swimming exercise enhances both postinjury axon regrowth as well as functional recovery. We further showed that the activity of the metabolic energy sensor AMPK in muscle as well as in injured neuron is critical in converting the energy spent during exercise into the positive effect in axon regeneration (Fig. 8).

In *C. elegans,* it is shown that a single swim session of 90 min presents exercise-like features such as muscle fatigue, a reduction of fat level in muscle, decrease in carbohydrate metabolism and enhanced mitochondrial oxidation (Laranjeiro et al., 2017). Many of these phenomena share commonalities with the physical exercise in vertebrate models (Thompson et al., 2001; Rivera-Brown and Frontera, 2012; Vina et al., 2012). In continuation to this finding, multiple and regular exercise sessions increase life-span, health quality, and protect against neurodegeneration in models of tauopathy, Alzheimer’s disease, and Huntington’s disease (Laranjeiro et al., 2019). It also increases overall learning ability as seen in other models and humans (Kobilo et al., 2014; Moon et al., 2016). These findings made it very relevant to address the role of physical exercise in promoting the repair of the injured neuronal circuitry using a worm model. We found that a 90-min swim session after the axotomy is sufficient to overcome age-related decline in axon regeneration and functional restoration. Intriguingly, this exercise paradigm is also sufficient to improve the functional recovery in early stages of life. Previous literature has demonstrated the benefit of physical exercise in peripheral nerve regeneration (Park and Höke, 2014; Gordon and English, 2016; Chen et al., 2017b). Since our behavioral assay is specific to single neuron responsible for touch sensation, we provided a direct evidence that enhanced axon regrowth because of physical exercise drives functional recovery.

An immediate effect of exercise is a reduction in the ratio of ATP/AMP (Chen et al., 2003), which is sensed by AMP Kinase (Hardie, 2011). Following the swimming session, we recorded a significant drop in ATP level. Therefore, we speculated a role of AMPK/AAK-2 in exercise-mediated improvement in touch neurons function. Consistent with this hypothesis, the loss of *aak-2* significantly abolished the improvement in touch neurons regeneration and function because of swimming exercise. Conversely, activation of AMPK using metformin was sufficient to promote axon regeneration. Tissue-specific rescue experiments suggested that AAK-2 acts both in neuron and muscle for enhanced axon regeneration and functional recovery. Few studies indicated that activation of AMPK by metformin has positive effect after spinal cord injury (Zhang et al., 2017; Guo et al., 2018). Pharmacological activation of AMPK can promote muscle fiber regeneration in a mouse myopathy model (Peralta et al., 2016). The AMPK agonist AICAR enhances spatial memory in wild-type animals, but this improvement was lacking in muscle-specific mutant of AMPK pointing toward the link between muscles and nervous system (Kobilo et al., 2014).

The question is how activated AAK-2 could be enhancing axon regeneration. It has been observed that postexercise activation of AMPK leads to the ATP synthesis via various metabolic pathways (Winder and Hardie, 1996; Hutber et al., 1997; Hardie, 2004). It also leads to mitochondrial biogenesis through proliferator-activated receptor γ coactivator-1α (PGC-1α; Zong et al., 2002; Kukidome et al., 2006). After a neuronal injury, the growth cone formation and subsequent regrowth is driven by various enzymes and motors. This requires high level of energy (Bradke et al., 2012; Zhou et al., 2016). Therefore, AMPK-driven ATP production can boost up the initiation of axon regeneration process as seen in our analysis. Apart from restoring the cellular energy levels, AMPK also regulates autophagy which allows the cell to survive the metabolic stress (Zhao and Klionsky, 2011). Autophagy induction has been linked with the stabilization of microtubules and enhancement of axonal regeneration after neuronal injury (He et al., 2016; Ko et al., 2020). Other possibility through which the AMPK can enhance axon regeneration is through DAF-16/FOXO1 activation. The activated AMPK can directly phosphorylate and regulate DAF-16 (Greer et al., 2007), which is known to regulate axon regeneration (Byrne et al., 2014). It would be an interesting direction to unravel how AMPK signaling couples muscle and neuron in neuronal regeneration.

## Synthesis

Reviewing Editor: Jeffery Twiss, University of South Carolina Decisions are customarily a result of the Reviewing Editor and the peer reviewers coming together and discussing their recommendations until a consensus is reached. When revisions are invited, a fact-based synthesis statement explaining their decision and outlining what is needed to prepare a revision will be listed below. The following reviewer(s) agreed to reveal their identity: Massimo Hilliard.

The reviewers had an overall positive view of your manuscript and noted that experimental design is largely solid and the results showing that physical exercise helps repairing nerve damage in C. elegans brings an advance. They also noted the non-cell autonomous effects of aak-2 and ATP or energy generation as quite interesting. The reviewers also noted that swimming in C. elegans leads to physiological changes that share similarities to those associated with physical exercise in mammals. Despite these strengths, the reviewers and reviewing editor felt that a number of issues need to be addressed prior to further consideration. I am appending the comments below, and I agree with the points that both raised. Addressing their major points will strengthen the manuscript. In particular, reviewer # 2’s points 1-4 should address the question of whether the functional recovery is due to a better axonal regrowth vs. improved maintenance of the distal axon/axonal fusion. I think it is appropriate to distinguish these two mechanisms here.

Reviewer 1:

This study tests the idea that physical exercise helps repairing nerve damage using C. elegans model. The overall experimental design is solid, built on two recent reported experimental paradigms- 1. swimming C. elegans leads to physiological changes that share similarities to those associated with physical exercise in mammals; 2. axotomized touch neuron axons can regenerate and regain mechanosensation. One interesting finding appears to be that aak-2 and ATP or energy affects axon regeneration cell non-autonomously.

The authors are to be commended for carrying out this series of experiments, as they are both time demanding and also require highly sophisticated skills in handling the animals. While the data collection and quantification analyses in axotomy of various conditions and genetic mutants are technically acceptable, the number of animals is mostly < 20, and it is unclear whether the complied data represent how many biological replicates.

The main issue is the writing-- it is often difficult to figure out from the main text exactly how animals were handled, what the exact genotype of the animals was. This should be addressed by including a complete data file as stated above. The manuscript should also be carefully edited, perhaps with the help of professional services.

For extended data, the authors should provide a table or excel file with the complete information on the full genotypes of animals and strain designation, as well as the exact experimental treatment-- swimming time, drug incubation time, Axotomy-induced loss of mechanosensation, and regeneration-associated gain of mechanosensation. It would be best to match the data file with each data graph in the Figures.

other specific points -

Line 282: the authors state that levamisole acts as a muscle relaxant, citing Culetto E, et al, 2004. It is unclear how this effect is achieved, as levamisole activates ACh receptors on the muscle, which causes muscle contraction.

Line 337: it is unclear why ‘P38’ is written with 38 superscript

Line 437: the authors state ‘...both touch neuron and muscle specific expression of aak-2 rescues the axon...’ does this experiment mean co-expressing aak-2 in touch neuron and muscle, or just either neuron or muscle?

Reviewer 2 Comments:

The manuscript entitled “Swimming exercise promotes post-injury axon regeneration and functional restoration through AMPK", characterizes the effect of exercise on the functional and morphological axonal recovery upon a severing injury. The authors focus on the well-defined C. elegans PLM mechanosensory neurons, and on the laser-induced axotomy as a surgical axotomy procedure. They reveal that a single set of 90 min swimming following axotomy has a beneficial effect on the restoration of the PLM mechanosensory neuron function and on the morphological regenerative modifications of the axon. They also identify that change in AMP/ATP levels is an important element in this exercise-mediated recovery process, revealing with genetic and pharmacological analysis the function of AMPK in this process. The manuscript is overall well developed and well written, with a comprehensive set of experiments and control to address the formulated hypothesis. There are however some conceptual elements and concerns that need to be addressed before the paper can proceed further.

Major points -

1. One element of concern is related to the functional recovery and how this is achieved.

In two back-to-back papers in PNAS, it has been shown the importance of axonal fusion for functional recovery (Basu et al., 2017; Abay et a., 2017). However, in this current manuscript with the swimming paradigm, functional recovery appears to be - at least partially - independent from axonal fusion. Given the mixed axon/dendrite nature of the PLM neurite, there is a real possibility that the beneficial effect of swimming, and the observed functional recovery, are related to a better “persistence” of the distal fragment, rather than a rewiring of the proximal fragment. Did the authors examine the distal fragment appearance (i.e. GFP intensity, thinning, fragmentation etc.) in these experiments and correlate it with recovery? This is needed and will clarify a key point.

2. Related to point 1, the non-cell-autonomous role of AAK-2 might suggest that there is an effect on the PLM axon coming from surrounding tissues. The PLM distal fragment has been shown to be “cleared” by the surrounding epidermis (Nichols et a., 2016). Have the authors tried to rescue AAK-2 using an epidermal-specific promoter?

3. Still related to the previous points: Without axonal fusion, which would re-establish the same postsynaptic contacts of the PLM neuron, a rewiring of the proximal PLM axon would need new synapses (or gap junctions) to be established with the postsynaptic targets in order to activate the behavioural response. Have the authors visualized the PLM presynaptic loci using RAB-3 or SNB-1 to determine if new sites are formed within the 24 hr period? This is critical for their model.

4. In Figure 5G, H and I, the authors present data related to axonal fusion. However, this event is based on the intact appearance of the distal fragment, and should be calculated as a percentage of those axons that have reconnected (Neumann and Hilliard, 2019). It is not clear from the data presented here that this is indeed how the authors have measured it.

5. In the following instances, the authors make claims that are not substantiated by the data:

- Line 343 related to figure 4C it is stated: “Similar observation was made when experiment was conducted at L4 stage (Figure 4C).” However, in this panel the mlk-1(0) was not tested. This statement needs to be revised to reflect the data.

- Line 301, “...that our exercise regimen can improve the recovery index significantly when axotomy was performed at L4, A1, A3, A4 and A5 stages (Figure 2A).” the effect on A5 is not significant in this panel, and the statement is incorrect. Revise by removing A5.

- Line 430, it is stated: “We noticed that there is an increase in the recovery index for both non-fusion and fusion related events at A3 stage”. However, in figure 7F this is not significant for the fusion related events. This needs to be clarified.

- The experiment with unc-54 present in the figure 1E is not mentioned in the text.

- Line 437-442. This section is confusing as the authors discussed data of A3 and L4 stages. In L4, there is rescue in the recovery index using the muscle promoter. In the A3, (Extended Figure 7.2) there is no rescue. This should be made clear in the text.

6. In panel 1F, why unc-54 is not included?

7. Line 63, add reference Abay et al., (i.e. accompanying paper to Basu et al., in PNAS, which also shows decline with age in C. elegans).

8. Line 371 missing citations for self-fusion (Neumann et al., 2011, 2015).

9. Figure 3D, is it possible that if the swimming session is done earlier (i.e. more than 24 hours before the test) that only 1 swim session is needed for A8 and A10? This would suggest that if exercise is done early enough, it can improve age-decline PTRI without having to do multiple swim sessions.

10. Lines 352-354, in the following statement: “These results confirm that exercise mediated increase in functional recovery involves regrowth from the injured proximal stump of the PLM neuron and subsequent rewiring into the functional circuit.” The authors have not shown that the cause of the functional recovery is due to rewiring (related to points 1-4). Please revise.

Minor points -

1. In panel 1C what the n means?

2. Line 58, remove “Eric” from the reference.

3. Line 134/135, awkward, consider revising.

4. Line 140 “Pasteur”.

5. In the method, separate number from their respective unit measures.

6. The authors need to clarify when they state that they have repeated the test twice, if this means that the n number of animals is split in two.

7. Panel 5E. Given these are discrete numbers of filopodia, the best representation would be median, with the stats being only between full or half values.

8. Line 101, (PLM) neuron, add Posterior Lateral Microtubule (PLM) neuron.

9. Line 159, total protein (mg/ml), change to total protein (mg)

10. Line 256, remove “:” from Results.

11. Line 263, (Figure 1A, .....(Lanranjeiro et al., 2017)), brackets not closed.

12. Line 308, remove “,” after significantly.

13. Line 337, change to either p38 or P38.

14. Line 392, change “minuit
