# Supplementary material for: Swimming Exercise Promotes Post-injury Axon Regeneration and Functional Restoration through AMPK
Source: eNeuro. 2021 Jun 15;8(3):ENEURO.0414-20.2021. doi: 10.1523/ENEURO.0414-20.2021 (PMC8211466; doi:10.1523/ENEURO.0414-20.2021)
Supplement: Extended Data Figure 1-2 — The PTRI values at 3 and 24 h postaxotomy at A3 stage. The recovery index values were obtained by normalizing the PTRI values at 24 h with respect to that at 3 h. The data are presented from groups, which underwent swimming session of varying duration (30–120 min) Download Figure 1-2, DOCX file. [file enu-eN-NWR-0414-20-s06.docx]

**Extended data Figure1-2:** The Posterior Touch Response Index (PTRI) values at 3 h and 24 h postaxotomy at A3 stage. The recovery index values were obtained by normalizing the PTRI values at 24 h with respect to that at 3 h. The data is presented from groups, which underwent swimming session of varying duration (30 min-120 min).

| **30 min**  **Non-Swimming control** | | |
| --- | --- | --- |
| **PTRI @ 3h** | **PTRI @ 24h** | **Recovery Index** |
| 0.6 | 0.6 | 1.0 |
| 0.6 | 0.6 | 1.0 |
| 0.5 | 0.5 | 1.0 |
| 0.6 | 0.6 | 1.0 |
| 0.5 | 0.6 | 1.2 |
| 0.5 | 0.6 | 1.2 |
| 0.6 | 0.5 | 0.8 |
| 0.6 | 0.5 | 0.8 |
| 0.6 | 0.6 | 1.0 |
| 0.6 | 0.6 | 1.0 |
| 0.6 | 0.6 | 1.0 |
| 0.5 | 0.5 | 1.0 |
| 0.5 | 0.4 | 0.8 |
| 0.6 | 0.6 | 1.0 |
| 0.8 | 0.5 | 0.6 |
| 0.7 | 0.7 | 1.0 |
| 1 | 0.6 | 0.6 |
| 0.8 | 1 | 1.3 |
| 0.7 | 0.8 | 1.1 |
| 0.4 | 0.7 | 1.8 |

| **30 min**  **Swimming group** | | |
| --- | --- | --- |
| **PTRI @ 3h** | **PTRI @ 24h** | **Recovery Index** |
| 0.6 | 0.6 | 1.0 |
| 0.6 | 0.6 | 1.0 |
| 0.6 | 0.6 | 1.0 |
| 0.6 | 0.5 | 0.8 |
| 0.6 | 0.5 | 0.8 |
| 0.6 | 0.6 | 1.0 |
| 0.6 | 0.5 | 0.8 |
| 0.6 | 0.6 | 1.0 |
| 0.5 | 0.5 | 1.0 |
| 0.5 | 0.6 | 1.2 |
| 0.6 | 0.4 | 0.7 |
| 0.5 | 0.6 | 1.2 |
| 0.5 | 0.5 | 1.0 |
| 0.8 | 0.8 | 1.0 |
| 0.6 | 0.8 | 1.3 |
| 0.7 | 0.7 | 1.0 |
| 0.8 | 0.6 | 0.8 |
| 0.7 | 0.6 | 0.9 |
| 0.5 | 0.5 | 1.0 |
| 0.8 | 0.4 | 0.5 |
| 0.6 | 0.5 | 0.8 |

| **45 min**  **Non-Swimming control** | | |
| --- | --- | --- |
| **PTRI @ 3h** | **PTRI @ 24h** | **Recovery Index** |
| 0.5 | 0.5 | 1.0 |
| 0.5 | 0.6 | 1.2 |
| 0.5 | 0.6 | 1.2 |
| 0.6 | 0.5 | 0.8 |
| 0.6 | 0.5 | 0.8 |
| 0.8 | 0.7 | 0.9 |
| 0.8 | 0.6 | 0.8 |
| 0.6 | 0.5 | 0.8 |
| 0.5 | 0.5 | 1.0 |
| 0.5 | 0.6 | 1.2 |
| 0.7 | 0.5 | 0.7 |
| 0.5 | 0.6 | 1.2 |
| 0.7 | 0.5 | 0.7 |
| 0.9 | 0.7 | 0.8 |
| 0.8 | 0.6 | 0.8 |
| 0.4 | 0.6 | 1.5 |
| 0.7 | 0.4 | 0.6 |
| 0.8 | 0.8 | 1.0 |
| 0.8 | 0.6 | 0.8 |
| 0.7 | 0.7 | 1.0 |
| 0.7 | 0.6 | 0.9 |
| 0.4 | 0.5 | 1.3 |
| 0.7 | 0.6 | 0.9 |

| **45min**  **Swimming group** | | |
| --- | --- | --- |
| **PTRI @ 3h** | **PTRI @ 24h** | **Recovery Index** |
| 0.5 | 0.5 | 1.0 |
| 0.6 | 0.7 | 1.2 |
| 0.6 | 0.8 | 1.3 |
| 0.5 | 0.6 | 1.2 |
| 0.5 | 0.6 | 1.2 |
| 0.6 | 0.7 | 1.2 |
| 0.6 | 0.7 | 1.2 |
| 0.7 | 0.6 | 0.9 |
| 0.4 | 0.5 | 1.3 |
| 0.5 | 0.5 | 1.0 |
| 0.6 | 0.5 | 0.8 |
| 0.4 | 0.5 | 1.3 |
| 0.6 | 0.6 | 1.0 |
| 0.6 | 0.6 | 1.0 |
| 0.8 | 0.5 | 0.6 |
| 0.8 | 0.8 | 1.0 |
| 0.8 | 0.7 | 0.9 |
| 0.6 | 0.8 | 1.3 |
| 0.7 | 0.7 | 1.0 |
| 0.5 | 0.7 | 1.4 |
| 0.6 | 0.8 | 1.3 |
| 0.7 | 0.7 | 1.0 |
| 0.6 | 0.6 | 1.0 |
| 0.5 | 0.7 | 1.4 |
| 0.8 | 0.7 | 0.9 |

| **90min**  **Non-Swimming control** | | |
| --- | --- | --- |
| **PTRI @ 3h** | **PTRI @ 24h** | **Recovery Index** |
| 0.6 | 0.6 | 1.0 |
| 0.6 | 0.7 | 1.2 |
| 0.5 | 0.6 | 1.2 |
| 0.6 | 0.6 | 1.0 |
| 0.6 | 0.5 | 0.8 |
| 0.8 | 0.7 | 0.9 |
| 0.6 | 0.6 | 1.0 |
| 0.6 | 0.6 | 1.0 |
| 0.7 | 0.6 | 0.9 |
| 0.6 | 0.5 | 0.8 |
| 0.5 | 0.5 | 1.0 |
| 0.5 | 0.5 | 1.0 |
| 0.5 | 0.7 | 1.4 |
| 0.7 | 0.7 | 1.0 |
| 0.8 | 0.4 | 0.5 |
| 0.6 | 0.6 | 1.0 |
| 0.8 | 0.7 | 0.9 |
| 0.7 | 0.9 | 1.3 |
| 0.8 | 0.8 | 1.0 |
| 0.8 | 0.8 | 1.0 |
| 0.2 | 0.4 | 2.0 |

| **90min**  **Swimming group** | | |
| --- | --- | --- |
| **PTRI @ 3h** | **PTRI @ 24h** | **Recovery Index** |
| 0.5 | 0.7 | 1.4 |
| 0.5 | 0.7 | 1.4 |
| 0.6 | 0.7 | 1.2 |
| 0.5 | 0.8 | 1.6 |
| 0.5 | 0.6 | 1.2 |
| 0.6 | 0.7 | 1.2 |
| 0.4 | 0.6 | 1.5 |
| 0.6 | 0.6 | 1.0 |
| 0.5 | 0.8 | 1.6 |
| 0.5 | 0.8 | 1.6 |
| 0.6 | 0.8 | 1.3 |
| 0.7 | 0.9 | 1.3 |
| 0.6 | 0.8 | 1.3 |
| 0.7 | 0.9 | 1.3 |
| 0.4 | 0.7 | 1.8 |
| 0.7 | 0.8 | 1.1 |
| 0.7 | 0.7 | 1.0 |
| 0.7 | 0.6 | 0.9 |
| 0.6 | 0.8 | 1.3 |
| 0.6 | 0.8 | 1.3 |

| **120 min**  **Non-Swimming control** | | |
| --- | --- | --- |
| **3 h PTRI** | **24 h PTRI** | **Recovery Index** |
| 0.5 | 0.5 | 1.0 |
| 0.6 | 0.7 | 1.2 |
| 0.6 | 0.6 | 1.0 |
| 0.6 | 0.5 | 0.8 |
| 0.6 | 0.5 | 0.8 |
| 0.5 | 0.6 | 1.2 |
| 0.5 | 0.5 | 1.0 |
| 0.5 | 0.5 | 1.0 |
| 0.6 | 0.6 | 1.0 |
| 0.5 | 0.5 | 1.0 |
| 0.5 | 0.6 | 1.2 |
| 0.5 | 0.4 | 0.8 |
| 0.5 | 0.5 | 1.0 |
| 0.5 | 0.4 | 0.8 |
| 0.6 | 0.6 | 1.0 |
| 0.4 | 0.5 | 1.3 |
| 0.5 | 0.4 | 0.8 |
| 0.7 | 0.8 | 1.1 |
| 0.8 | 0.5 | 0.6 |
| 0.6 | 0.8 | 1.3 |
| 0.7 | 0.6 | 0.9 |
| 0.5 | 0.6 | 1.2 |
| 0.7 | 0.7 | 1.0 |
| 0.6 | 0.6 | 1.0 |

| **120 min**  **Swimming group** | | |
| --- | --- | --- |
| **3 h PTRI** | **24 h PTRI** | **Recovery Index** |
| 0.5 | 0.6 | 1.2 |
| 0.4 | 0.7 | 1.8 |
| 0.5 | 0.7 | 1.4 |
| 0.6 | 0.7 | 1.2 |
| 0.5 | 0.6 | 1.2 |
| 0.5 | 0.8 | 1.6 |
| 0.4 | 0.6 | 1.5 |
| 0.5 | 0.7 | 1.4 |
| 0.6 | 0.6 | 1.0 |
| 0.5 | 0.7 | 1.4 |
| 0.5 | 0.6 | 1.2 |
| 0.5 | 0.8 | 1.6 |
| 0.5 | 0.8 | 1.6 |
| 0.6 | 0.9 | 1.5 |
| 0.5 | 0.5 | 1.0 |
| 0.5 | 0.4 | 0.8 |
| 0.7 | 0.6 | 0.9 |
| 0.7 | 0.8 | 1.1 |
| 0.6 | 0.7 | 1.2 |
| 0.6 | 0.8 | 1.3 |
| 0.7 | 0.7 | 1.0 |
| 0.7 | 0.7 | 1.0 |
